# Understanding the Effect of the Synthetic Method and Surface Chemistry on the Properties of CsPbBr_3_ Nanoparticles

**DOI:** 10.3390/nano14010081

**Published:** 2023-12-27

**Authors:** Mariangela Giancaspro, Annamaria Panniello, Nicoletta Depalo, Roberto Comparelli, Marinella Striccoli, Maria Lucia Curri, Elisabetta Fanizza

**Affiliations:** 1 Dipartimento di Chimica, University of Bari, Via Orabona 4, 70126 Bari, Italy; mariangela.giancaspro@uniba.it; 2 National Research Council (CNR)-Institute for Physical Chemistry Processes (IPCF), SO Bari, Via Orabona 4, 70126 Bari, Italy; a.panniello@ba.ipcf.cnr.it (A.P.); n.depalo@ba.ipcf.cnr.it (N.D.); m.striccoli@ba.ipcf.cnr.it (M.S.); 3 Consorzio Interuniversitario Nazionale per la Scienza e Tecnologia dei Materiali (INSTM), Bari Research Unit, 50121 Firenze, Italy

**Keywords:** surface passivation, CsPbBr_3_ nanoparticles, hot injection approach, ligand-assisted reprecipitation in polar solvent-free synthetic method

## Abstract

Over the last decade, the attractive properties of CsPbBr_3_ nanoparticles (NPs) have driven ever-increasing progress in the development of synthetic procedures to obtain high-quality NPs at high concentrations. Understanding how the properties of NPs are influenced by the composition of the reaction mixture in combination with the specific synthetic methodology is crucial, both for further elucidating the fundamental characteristics of this class of materials and for their manufacturing towards technological applications. This work aims to shed light on this aspect by synthesizing CsPbBr_3_ NPs by means of two well-assessed synthetic procedures, namely, hot injection (HI) and ligand-assisted reprecipitation (LARP) in non-polar solvents, using PbBr_2_ and Cs_2_CO_3_ as precursors in the presence of already widely investigated ligands. The overall goal is to study and compare the properties of the NPs to understand how each synthetic method influences the NPs’ size and/or the optical properties. Reaction composition and conditions are purposely tuned towards the production of nanocubes with narrow size distribution, high emission properties, and the highest achievable concentration. As a result, the formation of bulk crystals as precipitate in LARP limits the achievement of a highly concentrated NP solution. The size of the NPs obtained by LARP seems to be poorly affected by the ligands’ nature and the excess bromide, as consequence of bromide-rich solvation agents, effectively results in NPs with excellent emission properties. In contrast, NPs synthesized by HI exhibit high reaction yield, diffusion growth-controlled size, and less striking emission properties, probably ascribed to a bromide-deficient condition.

## 1. Introduction

Lead halide perovskites as thin films or nanoparticles (NPs), either organic-inorganic or all-inorganic, are promising materials [1,2] for many applications, as they exhibit outstanding optical properties, including a high extinction coefficient in the visible range, efficient photoluminescence, and narrow emission line widths [2], which makes them appealing as active components in solar cells [3,4,5] in photodetectors [6] and as photocatalysts [7,8,9]. However, the main limitations towards their large-scale production and commercialization have been mainly ascribed to their poor structural and thermal properties as well as poor photo-stability, all of which cause serious issues in material preparation, storage, application, and device fabrication [10,11]. Though improved thermal stability can be achieved by replacing the organic cation, like formamidinium or methylammonium, with inorganic caesium ions, issues related to PL quenching due to annealing or UV irradiation exposure and structural instability of the iodide cubic phase, which is the desired crystal phase for the photovoltaic properties, have still been reported [10]. Most of the reports aiming at overcoming stability issues have focused on CsPbBr_3_, which shows superior structural stability in ambient conditions [5], possesses a large bandgap of 2.3 eV, and demonstrates appropriate light-harvesting characteristics. Furthermore, owing to its excellent optoelectronic properties, narrow luminescence, and superior photoluminescence efficiencies thanks to its defect-tolerant electronic structure, CsPbBr_3_ possesses a unique potential for developing halide perovskite-based light-emitting diodes (LEDs), optically pumped lasers, luminescent solar concentrators, and anti-counterfeiting labels [12,13,14]. These material photophysical properties can be strongly enhanced by purposely selecting colloidal syntheses conditions. In particular, careful surface engineering is required to control passivation by ligand nature and density, which can offer an improved photoluminescence quantum yield but also guarantee effective charge injection and transportation in CsPbBr_3_ film fabricated for LED devices [15]. Furthermore, thermal and photo-stability, which are partially attributed to ligand loss, can be also improved by proper surface chemistry.

Since the first report on the hot injection (HI) synthesis of CsPbBr_3_ NPs [16], much progresses have been made in understanding the role played by surface chemistry [17,18,19,20,21,22,23,24,25] in dictating NP colloidal and structural stability as well as governing the NPs’ optical properties. Even though CsPbBr_3_ NPs are defect-tolerant [26], appropriate surface passivation is still necessary to obtain highly emitting and stable NPs, since CsPbBr_3_ NPs suffer instabilities caused by the dynamic and labile nature of both the inorganic core and the organic−inorganic interface.

The originally used oleyl amine (Olam)/oleic acid (OA) pair, which have been shown to lead to a labile oleylammonium oleate/bromide passivation layer, is subjected to a proton exchange reaction involving primary oleylammonium salts [27,28]. Such ligand couples have been replaced by quaternary alkyl ammonium bromide (e.g., didodecyl ammonium bromide, DDAB) [24,28], which completely lacks protons, granting better surface passivation [24], as DDAB is less prone to reacting with the surrounding environment. Further, alkyl phosphonic acid (PA) ligands have been used in synthesis, having been demonstrated to be beneficial for NPs’ stability. The PA can replace Br^−^ anions and strongly coordinate the Pb^2+^ sites by means of hydrogen phosphonate (PA^−^), phosphonate (PA^2−^), and phosphonic acid anhydride groups [29,30,31,32,33], thus affecting both nucleation and growth steps and surface passivation.

As highlighted by the density functional theory (DFT) calculations [23], the binding-energy values of all these ligands are similar (~50−60 kcal/mol for PA-Pb^2+^, 51.3 kcal/mol for Cs-oleate, 45.3 kcal/mol for primary alkylammonium-Br, and 48.2 kcal/mol for DDA-Br). Therefore, the difference in the ligands’ shell stability is derived mainly from the ligand–ligand and ligand–solvent interaction. Several works have, for example, verified that DDAB-capped NPs are more robust in a polar dispersant solvent due to the poor solubility of this ligand [24].

In addition to HI approaches, which need high temperatures and an inert atmosphere, typically achieved in a Schlenk line, syntheses carried out in air at room temperature have been investigated by applying the ligand-assisted reprecipitation (LARP) method. This strategy does not require conditions as stringent as HI, as it is performed under air and needs only basic wet chemistry tools. In addition, it is inherently scalable and far more appealing to industry from a cost, energy, and complexity perspective [34]. In the early LARP approaches, halide salts are dissolved stoichiometrically in a polar solvent, typically dimethylformamide or dimethyl sulfoxide, in the presence of ligands. Further, this solution is injected into a miscible solvent in which the salts are poorly soluble, thus creating a supersaturation condition that triggers NP crystallization, limited by ligands’ coordination to the NP surface [34]. Recently, room-temperature LARP approaches in a non-polar medium have been also reported as an interesting alternative to conventional LARP methods [18,35]. Here, precursor salts are preliminary decomposed in non-polar solvents by the addition of solvation agents and then mixed at room temperature under a supersaturation condition. This route allows for overcoming some of the intrinsic limitations of the conventional LARP method, i.e., a low NP concentration and final NP instability due to the residual presence of a high-boiling-point polar solvent.

Though great advances have been achieved in the synthesis of CsPbBr_3_ NPs, a close comparison of the role played by the synthetic approach on the resulting NP properties has not been fully elucidated yet. It is worth noting that each distinct synthetic method (i.e., HI or LARP in polar and polar solvent-free reaction medium) poses constraints such as the temperature condition, precursors, and the ligand nature and concentration, which have been recognized as crucial in controlling the shape and size and improving emission properties and structural stability, all essential for high-quality CsPbBr_3_ NPs [2].

Therefore, this work aims to provide a rational understanding of the growth and of the role of the ligands shell composition of CsPbBr_3_ NPs, synthesized either by the HI or polar solvent-free LARP procedure. The intrinsic requirements posed by each synthetic approach make not possible to follow identical preparation methods in terms of ligand composition and concentration. For the syntheses, starting from the same precursors, PbBr_2_ and Cs_2_CO_3_, different surfactants have been employed, namely, Olam in combination with OA or DDAB in the presence of OA or nonanoic acid/tetraoctyl ammonium bromide (TOAB) or phosphonic ligands, including octyl phosphonic acid (OPA) and trioctylphosphine oxide (TOPO), helping the precursors’ solubilization [36] and NP surface stabilization. A suitable combination of solvation and ligation agents has been selected for each synthetic approach to provide monodispersed cuboidal-like NPs with a high reaction yield, estimated in terms of NP concentration, and remarkable emission properties. A comprehensive characterization based on spectroscopic, morphological, and compositional analysis has been carried out to rationalize how the specific synthetic method and its operating conditions affect the NP size, optical properties, and reaction yield. The goal is to highlight the expected results in terms of size and optical properties of the NPs achieved by the two synthetic methods, HI and LARP. A comparison of the NP quality, such as emission properties, relative photoluminescence quantum yield and reaction yield, is also presented.

## 2. Materials and Methods

Materials. PbBr_2_ (98%), Cs_2_CO_3_ (Alfa Aesar, Haverhill, MA, USA, 99.9%), nonanoic acid (NA, Sigma Aldrich, St. Louis, MO, USA, technical-grade, 96%), oleic acid (OA, Sigma Aldrich, technical-grade, 90%), oleyl amine (Olam, Sigma Aldrich, technical-grade, 70%), didodecyl dimethylammonium bromide (DDAB, Sigma Aldrich, technical-grade, 98%), octylphosphonic acid (OPA, 98%), trioctylphosphine oxide (TOPO, Sigma Aldrich, technical-grade, 90%), tetraoctyl ammonium bromide (TOAB), anhydrous toluene (Sigma Aldrich, 99.8%), and ethyl acetate (EtAc, Sigma Aldrich, 99.8%) were used without any further purification.

CsPbBr_3_ nanoparticles synthesized by HI tuning surface chemistry. Three samples, labeled HI-NP_Olam_, HI-NP_DDAB_, and HI-NP_OPA-DDAB_, were synthesized by means of the HI approach. In a three-necked flask, 26 mg of PbBr_2_ (0.075 mmol) was dispersed in 2 mL of ODE in the presence of a suitable amount of ligation and solvation agents. HI-NP_Olam_ was prepared following the already reported procedure [37,38], and new protocols were developed for HI-NP_DDAB_ and HI-NP_OPA-DDAB_. PbBr_2_ was dissolved in ODE using 0.25 mL Olam (0.8 mmol) and 0.25 mL OA (0.8 mmol) for the synthesis of HI-NP_Olam_, 0.25 mL of OA (0.8 mmol) and 46 mg of DDAB (0.1 mmol) for the preparation of HI- NP_DDAB_, or 50 mg of OPA (0.3 mmol) and 0.5 g of TOPO (1.3 mmol) for HI- NP_OPA DDAB_. All the reaction flasks were put under vacuum at 100 °C and then under inert atmosphere conditions while raising the temperature up to 180 °C. At this stage, 0.1 mL of Cs-oleate, prepared by decomposing 75 mg of Cs_2_CO_3_ (0.2 mmol) in 2.5 mL of ODE in the presence of 0.4 mL of OA, was injected. The solution turned green after injection, and it was swiftly cooled down by immersing the reaction vessel in an ice bath. Unreacted precursors were removed by several successive cycles of purification, based on the addition of EtAc to the reaction medium (3:1 volumetric ratio), followed by centrifugation at 13,000× *g* and redispersion of the precipitate in toluene. To limit HI-NP_OPA-DDAB_ aggregation during the purification, 150 μL of DDAB solution in toluene at different concentrations was added to the reaction mixture prior the addition of EtAc, and the minimum amount suitable for guaranteeing the final NP colloidal stability was determined, which is nearly 0.05 M, corresponding to 0.008 mmol of DDAB.

The pellets recovered from the purification procedure were finally dispersed in 1 mL toluene for further morphological and spectroscopic characterization or dried under vacuum for thermogravimetric analysis.

CsPbBr_3_ nanoparticles synthesized by LARP tuning surface chemistry. Three samples, labeled LARP-NP_Olam_, LARP-NP_DDAB_, and LARP-NP_OPA-DDAB_, were synthesized by means of the LARP approach, following the procedure reported in Giancaspro et al. [18] and detailed in the Supplementary Materials, using Olam and OA, DDAB in the presence of OA and TOAB, or OPA mixed with TOPO and post addition of DDAB, respectively. After a preliminary step of thermal decomposition of the precursors at 100 °C, the synthesis was carried out in an open vial by pouring 55 μL of the Cs-oleate or Cs-nonanoate solution (both corresponding to 11 μmol of Cs) into 0.5 mL of the lead/halide precursor solution (0.1 mmol PbBr_2_). Details of the ligand compositions and the procedure used for the synthesis of each sample are reported in the Supplementary Materials. Purification was carried out by adding EtAc after 120 s of reaction in a 3:1 (EtAc: reaction batch) volume ratio using a two-step procedure, as described in Ref. [18].

The pellets recovered from the purification steps were finally dispersed in 1 mL toluene for the subsequent morphological and spectroscopic characterization or, alternatively, they were dried for thermogravimetric analysis.

Transmission electron microscopy (TEM). Carbon-coated copper grids were dipped in the NP colloidal solution diluted 1:20 with toluene, allowing the solvent to evaporate. TEM micrographs were acquired with a JEOL (Akishima City, Japan) JEM1011 electronic microscope operating at 100 kV, equipped with a high-resolution CCD camera. Statistical analysis of the lateral average size of the nanocubes was performed by Image J. The percentage relative standard deviation (σ%) was also calculated.

UV-Vis spectroscopy. UV-Vis absorption spectra of all CsPbBr_3_ NP samples were recorded using a Cary Varian (Cary, NC, USA) 5000 spectrophotometer equipped with a double detector. The absorption coefficient ε was calculated as reported by Maes et al. [39] according to the following equation:

$$\varepsilon =\frac{N_{A}\mu_{i}}{ln10}d^{3}$$

where $\mu_{i}$ is the intrinsic absorption coefficient ($\frac{N_{A}\mu_{i}}{ln10}$ = 0.0242), and d is the average diameter, as calculated from TEM analysis. The concentration of each sample was calculated based on the Lambert–Beer law, knowing the extinction coefficient (see Table S1), the absorbance, and the dilution factor.

Steady-state PL and time-resolved photoluminescence measurements. Steady-state photoluminescence (PL) spectra and time-resolved photoluminescence (TRPL) measurements were recorded for the CsPbBr_3_ colloidal solution with an optical absorption below 0.15 to avoid inner filter effects [40]. PL spectra were acquired by means of a HORIBA Jobin-Yvon (Palaiseau, France) Fluorolog 3 spectrofluorometer, equipped with double grating excitation and emission monochromators, using an excitation wavelength at 375 nm. TRPL measurements were carried out using the Time-Correlated Single-Photon Counting (TCSPC) technique using a picosecond laser diode (NanoLED HORIBA Scientific 375L, excitation at 375 nm), with a pulse length of 80 ps and 1 MHz repetition rate. The PL signals were dispersed by a double grating monochromator and detected by a picosecond photon counter (TBX Photon Detection Module, HORIBA Jobin-Yvon). The time resolution of the experimental set up was ~200 ps.

The relative PL quantum yield of the CsPbBr_3_ samples was estimated using Coumarin 153 in ethanol as a standard reference, including the correction for solvent refractive indices at 375 nm excitation wavelength, within the ratio calculation [40]. The PLQY of Coumarin 153 in ethanol is taken as 38% [41].

Thermogravimetric analysis (TGA). TGA was carried out using a Pyris 1-Perkin Elmer instrument (Shelton, CT, USA) under a nitrogen flow of 40 mL/min at a heating rate of 20 °C/min in a temperature range from 50 °C to 700 °C. Thermograms were collected using the powder of dried NP samples.

EDX analysis. Elemental analyses of the powders were performed by energy-dispersive X-ray analysis (EDX) on a Field Emission Sigma Zeiss SEM microscope (ZEISS, ΣIGMA, Hong Kong, China) equipped with a LaB_6_ source thermal field emitter and a Gemini objective lens. The samples for EDX characterization were prepared by drop casting the NP colloidal dispersion solutions onto an extensively washed silica substrate. The measurements were performed at a working distance of 7 mm and an electron gun voltage of 15 keV.

## 3. Results

Based on the reports on the syntheses of CsPbBr_3_ NPs, which highlight the advantages in stability and optical properties offered by ligand engineering [42], here, different combinations of surfactants, namely, Olam/OA, DDAB/NA/TOAB, DDAB/OA, or OPA/TOPO, have been used, and their relative molar ratio purposely tuned for the preparation of highly emissive CsPbBr_3_ NPs by either the HI or LARP approach. Surfactants in the reaction medium may act as a precursor solubilizer (i.e., TOAB and TOPO) and provide stabilizing agents for NPs. The obtained CsPbBr_3_ NPs can be considered to be made of a stoichiometric CsPbBr_3_ core, a PbBr_2_ “inner shell”, and an A′X′ “outer shell” [CsPbBr_3_](PbBr_2_){A′X′}, where A′ can be cesium ions, oleyl ammonium, or didodecyl dimethyl ammonium, and X′ can be oleate or bromide. PA derivatives usually bind the Pb^2+^ sites [23].

**Figure 1:**
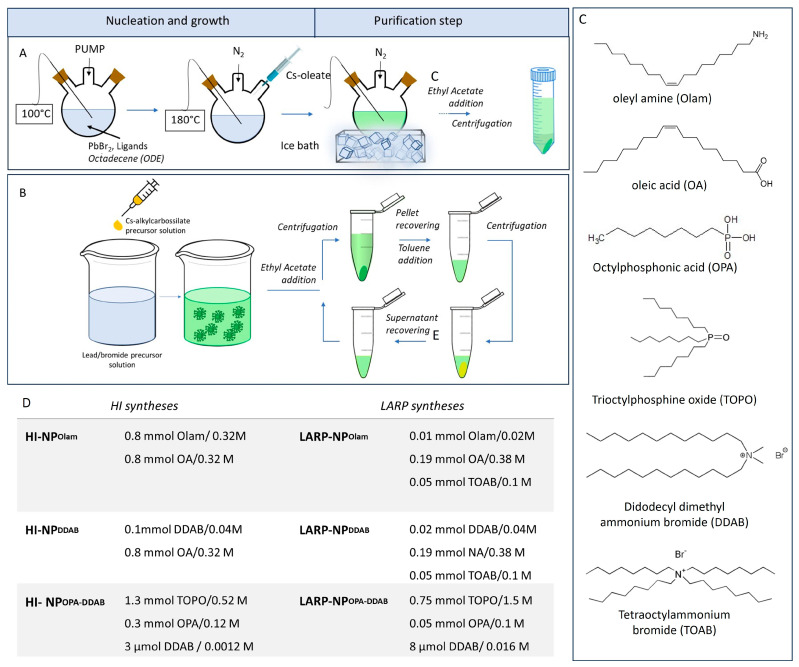
Schematic representation of the (**A**) HI and (**B**) polar solvent-free LARP methods used for the syntheses of CsPbBr_3_ NPs stabilized by different ligands (**C**). Panel (**D**) reports the amount and concentration of ligands and/or solvation agents used for each sample preparation. Includes 0.075 mmol of PbBr_2_ and a molar ratio of Pb^2+^: Cs^+^ of nearly 5:1. HI syntheses: volume 2 mL, LARP syntheses: volume 0.5 mL.

Figure 1A,B reports a schematic representation of the HI (Figure 1A) and polar solvent-free LARP (Figure 1B) procedures developed in this work for the synthesis of CsPbBr_3_ NPs.

HI syntheses have been carried out, fixing the injection temperature of the caesium-oleate precursor at 180 °C in the reaction mixtures of the lead/halide precursors. It has been reported that when an appropriate composition of surfactants is used, this temperature promotes the formation of a high yield of nanocubes, avoiding shape polydispersity [2].

The polar solvent-free LARP protocol has been applied following the procedure described by Giancaspro et al. [18]. It is preferred to the conventional LARP approach, since it allows for overcoming some of the critical issues associated with such a synthetic procedure. Indeed, an apolar solvent is used for the room-temperature NP crystallization, thus avoiding the use of a polar solvent with a high boiling point, which is hard to remove during the purification. Furthermore, the synthesis profits from a preliminary thermal decomposition step and the addition of solvation agents that help in the solubilization of larger quantities of salt precursors in the toluene used as the reaction medium.

The volume of reaction solvents, which is larger in the HI than in the LARP method (volume ratio 4:1), and, concomitantly, the use of the same amount of precursor, with lead bromide-exceeding caesium ions (PbBr_2_: Cs^+^ 5:1), result in an HI-NP reaction mixture more diluted in precursors than in LARP-NP. Such evidence agrees with the basic principles of the LARP approaches, featuring supersaturation conditions in the non-polar medium to promote the NP crystallization. Furthermore, the more diluted reaction mixtures in HI-NPs, along with the higher reaction temperature, are expected to limit aggregation phenomena.

The number of purification cycles has been purposely defined to effectively remove excess surfactants, side products, and unreacted precursors, meanwhile preserving colloidal stability and limiting detrimental loss of the sample as bulk precipitate due to ligand desorption during purification.

The composition of the reaction mixture, reported in Figure 1D, refers to the syntheses resulting in cuboidal NPs with enhanced optical properties, narrower size distribution, and higher reaction yield. A proper combination of conventional surfactants, like Olam and OA, has been used in the synthesis of the samples labelled NP_Olam_. DDAB with an excess of OA or NA is exploited in NP_DDAB_ and OPA mixed with TOPO, with post-synthesis addition of DDAB in the synthesis of NP_OPA DDAB_ samples (Figure 1C,D).

LARP-NP_Olam_ and LARP-NP_DDAB_ make use of a large amount of TOAB to extensively solubilize the PbBr_2_. For the synthesis of HI-NP_OPA DDAB_ and LARP-NP_OPA DDAB_, a combination of OPA/TOPO with post-synthetic addition of DDAB has been exploited to avoid the irreversible aggregation, reported when PAs with a long alkyl chain are used [29,30,31,32,33].

**Figure 2:**
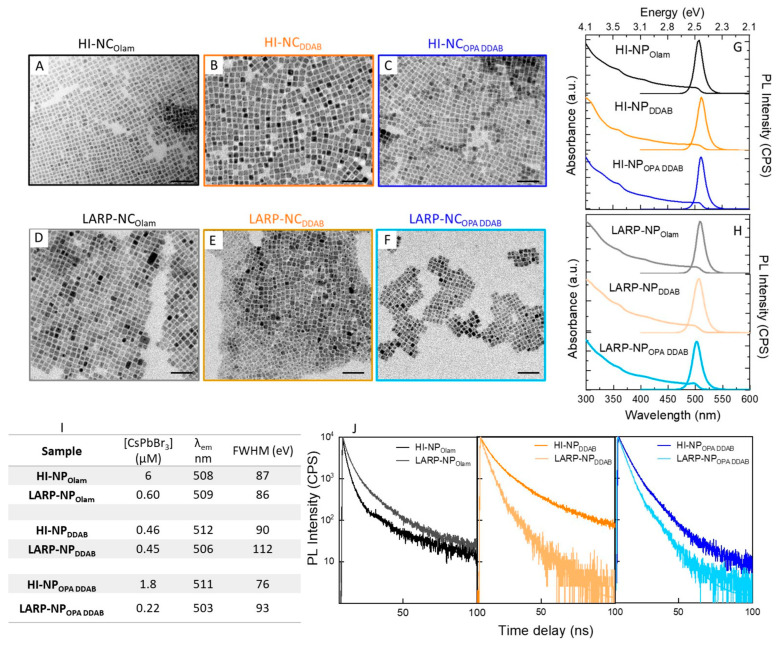
(**A**–**F**) TEM micrographs (scale bar 50 nm), (**G**–**H**) UV-Vis absorption and emission spectra (λ_ex_ = 400 nm), and (**J**) time-resolved photoluminescence decay of the CsPbBr_3_ colloidal nanoparticles synthesized by hot injection (HI-NP) and polar solvent-free ligand-assisted reprecipitation (LARP-NP) methods, in the presence of ligands and solvation agents, used in combination and at a molar ratio as reported in Figure 1D. In panel (**I**), the colloidal solution properties, reaction yield as expressed by micromolar concentration, emission wavelength (λ_em_), and full width at half maximum (FWHM) are reported. Color coding is used to identify the different samples.

**Figure 3:**
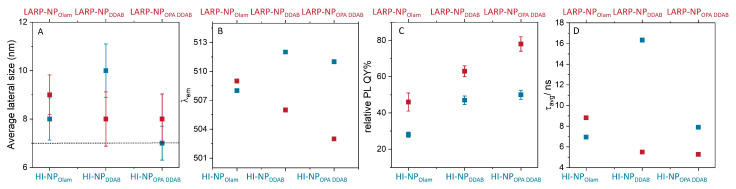
Scatter plots of (**A**) the statistical analysis of the nanocubes’ average lateral size and the size dispersion as determined from TEM micrographs, with the dashed line indicating the quantum confinement size for CsPbBr_3_, (**B**) the maximum wavelength of the PL emission, (**C**) the relative PL QY value with the standard deviation, and (**D**) the average lifetimes (τ_avg_) calculated by fitting the data in Figure 2J with a three-exponential decay function [40]. See Table S1 in Supplementary Materials for values and standard deviations.

The TEM and spectroscopic characterizations of the selected samples are reported in Figure 2, along with the detailed emission features, including the maximum wavelength of emission, λ_em,_ and full width at half maximum, FWHM, of the emission band, together with the calculated NP concentration value (table in Figure 2I, see also Supplementary Materials Table S1). It is worth noting that the NP concentration value (Figure 2) shows that the CsPbBr_3_ NP colloidal solutions obtained by the HI approach are more concentrated (in the micromolar range) than that achieved by LARP, which is an order of magnitude more diluted. A remarkably lower concentration (0.45 μM) is calculated from absorption measurements for HI-NP_DDAB_, resulting in the same range of the LARP-NP_DDAB_. Figure 3 displays the scatter plots, illustrating the average lateral size of the nanocubes (Figure 3A), λ_em_ of the PL peak (Figure 3B), relative PL QY (Figure 3C), and average PL lifetime (Figure 3D) for all the samples (See Table S1 in the Supplementary Materials).

Cuboidal NPs, with an average lateral size (Figure 2A–F and Figure 3A) of 8 nm (σ% = 11), 10 nm (σ% = 11), and 7 nm (σ% = 10) for HI- NP_Olam_, HI- NP_DDAB,_ and HI- NP_OPA DDAB_, respectively, and 9 nm (σ% = 9), 8 nm (σ% = 13), and 8 nm (σ% = 11) for LARP- NP_Olam_, LARP- NP_DDAB,_ and LARP- NP_OPA DDAB_, respectively, whose average size is not far from the quantum confinement size of CsPbBr_3_ (7.5 nm, Figure 3A), have been synthesized using the molar quantities reported in Figure 1D and performing two purification cycles. They are characterized by a typical absorption profile of CsPbBr_3_ (Figure 2G,H) and by a quite narrow emission band (<100 meV, Figure 2I) [23], which is even narrower for HI-NPs than LARP-NPs.

Considering the λ_em_ for each series of samples, the following trend can be observed: HI-NP_Olam_ (508 nm) < HI-NP_OPA DDAB_ (511 nm) ≤ HI-NP _DDAB_ (512 nm). This is only weakly related to the HI-NP size, which is HI-NP_OPA DDAB_ (7 nm) < HI-NP_Olam_ (8 nm) < HI-NP _DDAB_ (10 nm). Even though LARP-NPs show almost the same size (9–8 nm), the λ_em_ red shifts as follows: LARP-NP_OPA DDAB_ (503 nm) < LARP-NP_DDAB_ (506 nm) < LARP-NP_Olam_ (509 nm). These results are consistent with the poor quantum confinement of the NPs, thus resulting in a weak dependence of the emission maximum (λ_em_, Figure 3B) expected on their size (Figure 3A). In the literature, the position of the PL peak maximum has been also found to depend on NP concentrations. Though slight differences in the concentration of the NP colloidal solutions used for the PL characterization have been determined (see Table S1), a clear relationship with the λ_em_ position cannot be identified. It is worth noting that the concentration range of all solutions used for the PL characterization, estimated to be in the range of 5 nM–15 nM, is far below that which could induce an autoabsorption effect [43], which can also affect the position of the λ_em_. The results suggest that the discrepancy in the λ_em_ position can be sought in the different surface chemistry, originating from reaction mixture composition and synthetic condition and methods [23].

The relative PL QY reported in Figure 3C generally increases moving from NP_Olam_ to NP_DDAB_ and to NP_OPA DDAB_, irrespective of the synthetic approach, with the PL QY value obtained for LARP-NPs being much larger than those measured for HI-NPs, reaching almost 80% for LARP-NP_OPA DDAB_. The average lifetimes (τ_avg_), calculated by best-fitting the TRPL decay with a three-exponential function [40], are reported in Figure 3D. The slowest decay value, with τ_avg_ of nearly 16 ns for HI-NP_DDAB,_ and conversely, the fastest decay values (τ_avg_ nearly 5 ns), for LARP-NP_DDAB_ and LARP-NP_OPA DDAB,_ have been obtained.

The characterization carried out on the different CsPbBr_3_ samples confirms the clear dependence of the NP size and optical features on the nature and composition of ligation/solvation agents. Furthermore, the comparison of the properties of the samples prepared using similar surfactants but different synthetic approaches, HI or LARP, highlights a key role of the specific synthetic methodology in influencing the NP properties.

Now, to assess whether the ligands play a distinct role in each of the two synthetic approaches, the specific surface passivation resulting from each synthetic methodology has been studied. The organic and inorganic composition of CsPbBr_3_ NPs for each purified sample has been investigated by thermogravimetric analysis (TGA) and complementary energy-dispersive X-ray (EDX), with the former being informative for the organic contribution, whereas the latter is able to estimate the inorganic one.

From fundamental knowledge, surfactants, which are known to stabilize monomers and NPs, control nucleation and growth and affect surface passivation. A densely packed ligand shell, poorly soluble in the dispersion medium, has been shown to provide strong passivation, which is able to stabilize the NP’s surface. Also, the use of a reaction medium containing excess bromide results in NPs with superior CsPbBr_3_ emission properties, thanks to the reduction in halogen vacancies promoted by bromide adsorption [44,45].

**Figure 4:**
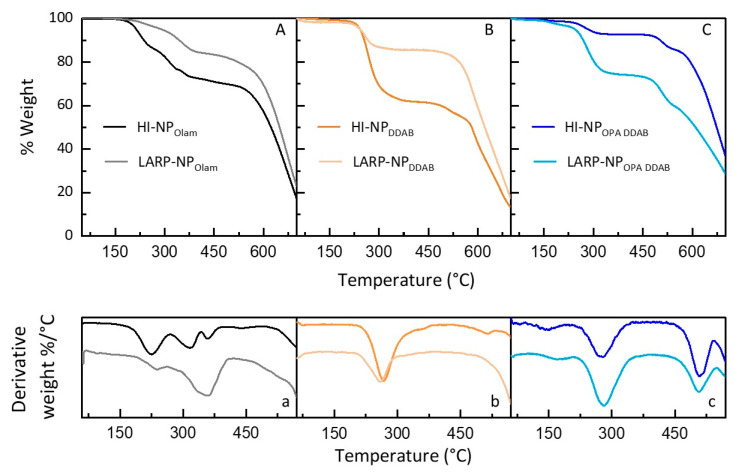
(**A**–**C**) Thermogravimetric and (**a**–**c**) first-derivative curves of NP_Olam_, NP_DDAB_, and NP_OPA DDAB_ synthesized by hot injection (HI) and polar solvent-free ligand-assisted reprecipitation (LARP) methods in the presence of ligation/solvation agents’ composition.

TGA has been performed under nitrogen flow on each NP sample collected as a pellet after purification and dried at 50 °C, by applying a heating ramp from 50 °C to 700 °C. TG and first-derivative (DTG) curves are reported in Figure 4. Above 550 °C, the weight loss can be reasonably ascribed to the CsPbBr_3_ decomposition [46,47], and the thermal events in the 50–550 °C range arise from degradation of the organic molecules, either weakly or tightly bound to NPs or free in solution. TGA can provide qualitative and quantitative information on the composition of the NP organic ligands by a comparison of the TG profile with those of pure ligands and solvation agents [48,49] used as references (See Table S2 in the Supplementary Materials) [18]. It is worth noting that the evaporation of ligands chemically bound to the NP surface results in weight losses at high temperatures and in a typical broadening of the TG profile [48,50].

As shown in Figure 4, a total weight loss of 27 wt% compared to 16% (Figure 4A) is attained for HI-NP_Olam_ with respect to LARP-NP_Olam_, whereas 35 wt% and 13% (Figure 4B) are achieved for HI-NP_DDAB_ and LARP-NP_DDAB_, respectively, and finally, HI-NP_OPA DDAB_ and LARP-NP_OPA DDAB_ show nearly 17 wt% and 37 wt%, respectively. The % weight loss remains almost unchanged for HI-NP_DDAB_ after the first cycle of purification (see Supplementary Materials Figure S1), revealing that more organic material is not removed by successive purification cycles. Two steps of purification of HI-NPs (see Supplementary Materials Figure S1) as well as for LARP-NPs [18] have been confirmed to be ideal for an efficient removal of residual ligands or solvation agents in the final NP solution, while still preserving NP colloidal stability.

The higher percentage value of weight loss for HI- NP_Olam_ and HI-NP_DDAB_ than for the LARP-NP counterparts can be related either to more densely packed surface-passivating organic shell or to the large amount of residual free ligands and solvation agents. A large amount of ligation and solvation agents have indeed been used in the reaction medium in HI synthesis.

NP_Olam_ weight losses occurred in two temperature ranges: a first one between 175 and 263 °C and a second range covering 265–400 °C. Even though the TG profile does not allow for discrimination between OA and Olam, the weight loss at a lower temperature could be associated with the elimination of free or physically adsorbed ligands, whereas that at a higher temperature can be ascribed to the evaporation of ligands bound to the surface of the NPs [48]. The TGA and DTG profiles suggest that a higher amount of unbound Olam and OA ligands is left in the colloidal solution of the HI-NP_Olam_, whereas in the LARP-NP_Olam_ samples, ligands are mostly at the NP surface. It is worth noting that TOAB, used to solubilize the lead/halide precursors, is expected not to bind the NP surface due to its high steric hindrance.

A single weight loss event in the range from 225 °C to 280 °C associated with the loss of the DDAB bound to the NP surface, with a peak at 260 °C, is measured for HI-NP_DDAB_ and LARP-NP_DDAB_. The % weight loss of nearly 35% for HI-NP_DDAB_, larger than the 13% of weight loss measured for LARP-NP_DDAB_, suggests a more densely packed ligand shell for the former samples.

Weight losses over the ranges of 150–200 °C (3% and 4%), 228–330 °C (7% and 25%), and 475–530 °C (7% and 12%) are shown for HI-NP_OPA DDAB_ and LARP-NP_OPA DDAB_, respectively, ascribed to the evaporation of NA or OA, DDAB, and TOPO/OPA, respectively [33]. A lower content of organic molecules forming the passivating organic shell or arising from residual excess in the colloidal solution is demonstrated for the HI-NP_OPA DDAB_. The TGA of HI-NP_OPA DDAB_ at different purification cycles (See Figure S1 in the Supplementary Materials) shows a large decrease in % weight loss corresponding to DDAB after the second cycle of purification, whereas the weight loss ascribed to OPA remains almost unchanged. Remarkably, in the HI-NP_OPA DDAB_ synthesis, DDAB is added to the reaction mixture right after the purification at room temperature, to prevent irreversible aggregation. Although the same addition is performed for LARP-NP_OPA DDAB_, a higher amount of residual DDAB is retained in the LARP-NP_OPA DDAB_ sample (25%). This percentage of weight loss is surprisingly even higher than that estimated for the LARP-NP_DDAB_, prepared by using a larger DDAB amount, thus suggesting a different extent of the DDAB coordination in the two samples. The shift of the weight loss at a higher temperature in the LARP-NP_OPA DDAB_ (170 °C with respect to 150 °C for HI-NP_OPA DDAB_), attributed to the NA, lets us suppose that NA is bound to the NP surface in this sample.

A Cs: Pb: Br atomic ratio of 0.7:1:5 for LARP-NP_Olam_, 1.4:1:6 for LARP-NP_DDAB_, and 1.5:1:7 for LARP-NP_OPA DDAB_ are calculated from EDX analysis. The resulting Br/Pb ratio > 3 appears to be consistent with bromide-rich synthetic conditions. Although a formal excess of PbBr_2_ over cesium has been always used in the synthesis, LARP-NP_DDAB_ and LARP-NP_OPA DDAB_ show a Cs/Pb ratio slightly higher than 1, and cesium-deficient stoichiometry is calculated for NP_Olam_. Therefore, CsBr-terminated CsPbBr_3_ NPs [51] can be assumed for NP_DDAB_ and NP_OPA DDAB_, with cesium being partially replaced by oleyl ammonium ions for NP_Olam_ [52,53]. Indeed, CsBr termination has already been demonstrated for cuboidal CsPbBr_3_ nanocrystals, with a more thermodynamically favored surface for NPs in the size range between 7 and 11 nm [51]. In fact, within this size regime, PbBr_2_ termination is unlikely to occur, as it would require much denser ligand packing, and hence experience a significant steric hindrance, with the consequent disruption of the Pb^2+^ octahedral coordination [51].

A Cs: Pb: Br atomic ratio of 1.1:1:3.1 for HI-NP_Olam_, 0.7:1:4 for HI-NP_DDAB_, and a 0.7:1:2.5 HI-NP_OPA DDAB_ are determined. Hence, a stoichiometry in agreement with CsPbBr_3_ is estimated for HI-NP_Olam_. In HI-NP_DDAB_, part of the caesium is expected to be replaced by DDA^+^, which is present in excess in the solution, as demonstrated by the TGA, whereas a bromide-rich synthetic condition results in a stoichiometric excess of bromide. For HI-NP_OPA DDAB_, the apparent CsBr-deficient stoichiometry can indeed arise from CsPbBr_3_ characterized by an outer PbBr_2_ shell, which has been already reported for CsPBbr_3_ synthesized in the presence of PA [23].

## 4. Discussion

The complementary techniques exploited to characterize and compare the HI-NPs and LARP-NPs can be leveraged to gain a clear understanding of the role played by the synthetic conditions set by HI and LARP, i.e., the reaction temperature in combination with the composition of precursors and surfactants, towards the control of the reaction yield and NP size, surface chemistry, and optical properties.

In the HI-NP syntheses, carried out at 180 °C, the ligands’ steric hindrance controls the NP size, concentration, and surface passivation. It has been demonstrated by TGA analysis that oleyl ammonium oleate, DDA^+^, and OPA/TOPO together with DDA^+^ stabilize the HI-NP_Olam_, HI-NP_DDAB,_ and HI-NP_OPA DDAB_, respectively.

HI-NP_Olam_ shows a higher reaction yield and good optical properties, thus supporting the relevant use of the Olam/OA pair in the HI synthesis of NPs [48]. However, the nearly stoichiometric bromide content, revealed by EDX analysis, can account for the low PL QY value measured for this sample, supported by the possibility of the occurrence of halide vacancies.

When DDAB is used in the synthesis to replace Olam, a slower diffusion from the bulk to the NP surface is expected, due to its steric hindrance. Larger nanostructures are hence formed, along with bulky precipitate, which is discarded, finally resulting in a low concentration of NPs. However, the stability of DDAB binding to NPs’ surface in toluene and its capability to fit the cesium sites results in a highly dense passivating shell, as shown by TGA, with part of the cesium ions replaced by DDA^+^, as revealed by cesium deficiency in EDX. Bromide excess is also estimated. Such surface chemistry contributes to enhancing the emission properties, as confirmed by the good relative PL QY (>40%) and the long τ_avg_ featured by the HI-NP_DDAB_ samples [23,54].

Conversely, smaller nanocubes and highly concentrated solution are achieved for HI-NP_OPA DDAB_. The strong coordination of phosphonate or anhydride ligands to Pb^2+^ sites limits monomer deposition in synthesis, resulting in nanocubes with a smaller average lateral size, and it avoids aggregation phenomena, preserving a high NP concentration even after purification. However, in comparison with the HI-NP_DDAB_, the faster τ_avg_ and similar PL QY suggest that excited states are less stabilized by surface passivation.

Absorption and emission spectra of the samples (See Figure S2 in the Supplementary Materials) have been recorded upon dilution to further investigate the ligand shell stability in HI-NP samples. Though the emission spectrum of the sample HI-NP_Olam_ shifts a few nanometers upon progressive dilution, because of partial ligands or inorganic fragment desorption, the emission spectra of HI-NP_DDAB_ and HI-NP_OPA DDAB_ samples do not change their position and full width at half maximum, suggesting that the passivation shell is stable upon the addition of toluene, probably due to poor ligand–solvent interactions. Concomitantly, absorption profiles remain unchanged for HI-NP_DDAB_ and HI-NP_OPA DDAB_. The observed stability against dilution can be ascribed to poor ligand−solvent interactions, rather than to the ligands’ binding affinity, as suggested by DFT calculation [23]. Therefore, the solubility of DDA-Br or Pb-phosphonates in toluene binding the CsPbBr_3_ surface, which is lower than that of the Cs-oleate or oleyl ammonium-Br, limits their detachment upon dilution, finally stabilizing HI-NP_DDAB_ and HI-NP_OPA DDAB_ against dilution more than HI-NP_Olam_.

In the LARP synthesis, the crystallization process, induced by supersaturation, is mainly controlled by reaction rather than by diffusion. Indeed, in this case, NP size seems less affected by the ligand composition, since, irrespective of the used surfactants, LARP-NP samples always feature NPs with a similar average lateral size (8–9 nm). Concomitantly, NPs are obtained at a lower concentration due to the easy occurrence of aggregation phenomena, with a high fraction of reaction products discarded during purification.

The large amount of TOAB used in LARP-NP_Olam_ and LARP-NP_DDAB_ results in a reaction mixture exhibiting an excess of bromide. TOAB, being a bulky alkyl ammonium bromide, is expected not to bind the NP surface; however, it could act as source of bromide, which strongly binds to the NP surface, competing with the organic ligand in the surface passivation. Such a description is confirmed by the results of the TGA, which shows a lower content of organic molecules for LARP-NP_Olam_ and LARP-NP_DDAB_ than for HI-NP counterparts, as well as EDX analysis, which reveals an excess of bromide. Conversely, the LARP-NP_OPA DDAB_ sample synthesized without TOAB, obtained upon post-synthesis DDAB addition, reveals a higher amount of DDAB, along with phosphonic derivatives, according to the TGA. This has also been confirmed by our previous study, where Nuclear Magnetic Resonance analysis suggests that phosphonic anhydride and DDA-Br bind to the NPs’ surface [18].

The bromide excess used for LARP-NP synthesis can account for the enhancement of the emission properties of LARP-NP samples compared to HI-NPs. Higher PL QY and, concomitantly, faster fluorescence decay have been measured for LARP-NP_DDAB_ and LARP-NP_OPA DDAB_. From these considerations, the use of solvation agents based on bromide-rich molecules such as TOAB in LARP synthesis turns out to be critical in defining the LARP-NP surface chemistry and the enhanced emission properties.

However, apart from an enhancement of the emission properties, excess bromide also results in the in situ formation of promoblumbates species prior to the cesium injection, which contribute to a poor control of the size monodispersity, mainly for LARP-NP_DDAB_, as fully described in our previous paper [18]. Conversely, a narrow size distribution is exceptionally achieved in HI thanks to thermodynamic and kinetic control of nucleation/growth, due to factors arising from reaction and composition conditions.

## 5. Conclusions

Cuboidal CsPbBr_3_ NPs have been synthesized by HI and LARP approaches in polar solvent-free reaction medium, employing widely used ligands based on primary alkyl ammine, like Olam in combination with alkyl carboxylic acid, quaternary alkyl ammonium bromide, or alkyl phosphonic acid, and well-established protocols. After determining the optimal reaction mixture for each synthetic approach, the ensuing properties including size, emission properties, concentration, and surface chemistry of the produced NPs have been discussed and rationalized with reference to the synthetic method. However, it is important to note that a direct comparison between HI and LARP based on identical mixture compositions and experimental conditions has not been intended in this work. The comprehensive characterization has highlighted that a supersaturation condition in LARP brings an undesirable loss of materials due to the formation of bulk nanostructures, which finally results in low a NP reaction yield compared to the HI method. Whereas HI syntheses seem to be controlled by diffusion, with surfactant composition affecting the NP size, LARP is mainly governed by the reactivity of precursors. This evidence is particularly highlighted when sterically hindered surfactants like DDAB are used, resulting in bigger NPs in HI-NP_DDAB_ sample than LARP-NP_DDAB_. Moreover, LAPR-NP_DDAB_ takes advantage of the large excess of TOAB, which provides a higher amount of bromide that guarantees passivation of halide-vacancies even though leading to a less dense DDAB passivation layer. Conversely HI-NP_DDAB_, synthesized at lower bromide content result in large amount of DDAB finally bound to HI-NP_DDAB_ surface, that partially replaces caesium sites, also providing a good and stable passivation shell. The combination of OPA/TOPO and DDAB, along with the bromide-rich condition, enable the easier preparation of nanocubes by LARP with greatly enhanced emission properties, with a relative photoluminescence quantum yield of almost 80% and fast fluorescence lifetimes.

This rational investigation of the properties of CsPbBr_3_ NPs and their comparison with respect to synthesis methodologies provides a useful tool to effectively drive the selection of the most suitable synthetic approach based on the desired properties of the materials, in view of their possible subsequent application.

## Data Availability

Data are contained within the article and supplementary materials.

## Supplementary Materials

The following supporting information can be downloaded at: https://www.mdpi.com/article/10.3390/nano14010081/s1, where the synthetic procedures for the preparation of LARP-NPs are reported, Table S1: Data corresponding to the average lateral size, relative PL QY, and average lifetimes (τ_avg_) and standard deviation; Table S2: Evaporation temperatures onset for all the ligands and solvation agents used in synthesis; Figure S1: Thermogravimetric analysis of the samples synthesized by hot injection after each purification cycle; Figure S2: UV-Vis absorption and emission spectra (λex = 375 nm) of HI-NP samples upon progressive dilution.
