# Supplementary material for: Understanding the Effect of the Synthetic Method and Surface Chemistry on the Properties of CsPbBr3 Nanoparticles
Source: Nanomaterials (Basel). 2023 Dec 27;14(1):81. doi: 10.3390/nano14010081 (PMC10780980; doi:10.3390/nano14010081)
Supplement: Supplementary file 1 [file nanomaterials-14-00081-s001.zip › nanomaterials-2761252-supplementary.pdf]

Supplementary Materials

Understanding the Effect of the Synthetic Method and Surface Chemistry on the Properties of $CsPbBr_3$ Nanoparticles

Mariangela Giancaspro [1,2], Annamaria Panniello [2], Nicoletta Depalo [2], Roberto Comparelli [2,3], Marinella Striccoli [2,3], Maria Lucia Curri [1,2,3,*] and Elisabetta Fanizza [1,2,3,*]

[1] Dipartimento di Chimica, University of Bari, Via Orabona 4, 70126 Bari, Italy; mariangela.giancaspro@uniba.it
[2] National Research Council (CNR)-Institute for Physical Chemistry Processes (IPCF), SO Bari, Via Orabona 4, 70126 Bari, Italy; a.panniello@ba.ipcf.cnr.it (A.P.); n.depalo@ba.ipcf.cnr.it (N.D.); m.striccoli@ba.ipcf.cnr.it (M.S.)
[3] Consorzio Interuniversitario Nazionale per la Scienza e Tecnologia dei Materiali (INSTM), Bari Research Unit, 50121 Firenze, Italy
* Correspondence: marialucia.curri@uniba.it (M.L.C.); elisabetta.fanizza@uniba.it (E.F.)

Organic ligand-capped $CsPbBr_3$ nanoparticles synthesized by ligand-assisted reprecipitation method.

Caesium precursor solution. Cs_2CO_3 (32.6 mg, 0.1 mmol) was dissolved in 1 ml of OA (3 mmol) or NA (5.6 mmol). The solution was heated at 80°C in open air and stirred for 1 hour prior to its use. The Cs-oleate (0.2 M) and Cs-nonanoate (0.2 M) precursor solution was used for the synthesis of $CsPbBr_3$ NPs.

Synthesis and purification of $CsPbBr_3$ LARP-NP_{Olam}. A lead/bromide precursor solution was prepared by adding 165.2 mg (0.45 mmol) of $PbBr_2$, 330 mg (0.6 mmol) of TOAB and 360 µL (1.15 mmol) of OA to 3 ml of toluene resulting in $[PbBr_2]$ = 0.13 M, [TOAB] = 0.18 M, [OA]=0.34 M. A second precursor solution was prepared by adding the same amount of $PbBr_2$ and OA, cutting to half the TOAB final concentration, 0.09 M. The precursor solution was heated at 70°C for 15 min. For the synthesis of $CsPbBr_3$ NPs, 34 µL of a solution of Olam in toluene (0.3 M, 0.01 mmol) were added to 0.5 mL of each $PbBr_2$ precursor solution, followed by the injection of 55 µL of the Cs-oleate solution (11 µmol) under vigorous stirring at room temperature. After further 120 s, EtAc was added as non-solvent to precipitate the NPs, with a reaction mixture: EtAc 1:6 v/v ratio, and subsequently the NPs were collected after each cycle of centrifugation/redispersion in toluene consisting in centrifugating at 10000 rpm for 10 minutes; the supernatant was discarded, and finally the precipitate was redispersed in 1 mL of toluene. These samples were labelled LARP-NP_{Olam}.

Synthesis and purification of $CsPbBr_3$ LARP-NP_{DDAB}. The lead/bromide precursor solution was prepared by adding 165.2 mg (0.45 mmol) of $PbBr_2$, 200 µL NA (1.15 mmol) and 330 mg (0.6 mmol) of TOAB to 3 ml of toluene, resulting in $[PbBr_2]$ = 0.14 M, [TOAB] = 0.09 M, [NA]=0.36 M. The precursor solution was heated at 70°C for 15 min. For the synthesis of the NPs, 10 mg (21 µmol) of DDAB were added to 0.5 ml of the lead/bromide precursor solution. The mixture was stirred at room temperature until DDAB completely dissolved. Then, 55 µL of the Cs-nonanoate solution (11 µmol) was quickly injected under vigorous stirring

and the solution was let stirring for 300 s. The two-step purification procedure was used to recover the NPs: by adding EtActo the reaction medium with a 3: 1v/v ratio and centrifuging at 13000g for 10 minutes; the supernatant was discarded, while the precipitate was redispersed in 100 µL of toluene, followed by a second step of centrifugation at 6000g. At this stage the supernatant was recovered for the second purification step. Finally, the pellet was redispersed in 1 mL of toluene and finally dispersed in 1 mL of toluene. These samples were labelled LARP-NP_{DDAB}.

Synthesis and purification of $CsPbBr_3$ LARP-$NP_{OPA\ DDAB}$. The synthesis was carried out according to the procedure reported by Brown et al. [1] with minor modifications. $PbBr_2$ (165.2 mg, 0.45 mmol) was dissolved in toluene (3 mL) in the presence of TOPO (1.7 g, 4.5 mmol) and the flask was heated at 70°C for 15 min prior to the addition of OPA (58 mg, 0.3 mmol). The precursor solution results in [$PbBr_2$] = 0.15 M, [TOPO] = 0.15 M, [OPA]=0.1 M. Cs-nonanoate solution (55 µL, 11 µmol) was injected in 0.5 mL of the lead/bromide precursor solution. After 30 s, 150 µL of a DDAB solution in toluene (0.05 M, 0.008 mmol) were added to the reaction mixture, and after further 300 s, the NPs were recovered by the addition of EtAc. A two-step and one-step purification procedures were performed. These samples were labelled LARP-$NP_{OPA\ DDAB}$.

Sample	Averge lateral size nm	σ (%)	λ_{em} nm	PLQY (%)	<τ> (ns)	ε ($cm^{-1}\mu M^{-1}$)	Molarity µM
**HI-NP$_{Olam}$**	8	11	508	28±2	6.94±0.08	12,3904	0,008
**LARP-NP$_{Olam}$**	9	9	509	46±5	8.82±0.15	17,6418	0,006
**HI-NP$_{DDAB}$**	10	11	512	47±2	16.34±0.08	20,74848	0,005
**LARP-NP$_{DDAB}$**	8	14	506	63±3	5.50±0.12	12,3904	0,008
**HI-NP$_{OPA\ DDAB}$**	7	10	511	50±3	7.9±0.06	8,3006	0,014
**LARP-NP$_{OPA\ DDAB}$**	8	13	503	78±4	5.27±0.04	12,3904	0,010

Table S1. Average lateral size, percentage of relative standard deviation (σ%) of the nanocubes as determined by statistical analysis of TEM micrographs, relative PL QY value and average lifetimes (τ_{avg} , <τ>) calculated by fitting the data in Figure 2J in the main manuscript with a three exponential decay function, extinction coefficient (ε) values and concentration of the colloidal solution used for the emission properties analysis.

Reference	T/°C
Olam	240
OA	280
NA	165°C
TOAB	220
DDAB	256
TOPO	248
OPA	384/504

Table S2 Evaporation temperature onset for pure ligands Olam= oleyl amine, OA= oleic acid, NA=nonanoic acid, TOAB= tetraoctyl ammonium bromide, DDAB= dimethyl didodecyl ammonium bromide, TOPO= trioctyl phosphine oxide, OPA= olctyl phosponic acid, as reported in literature.

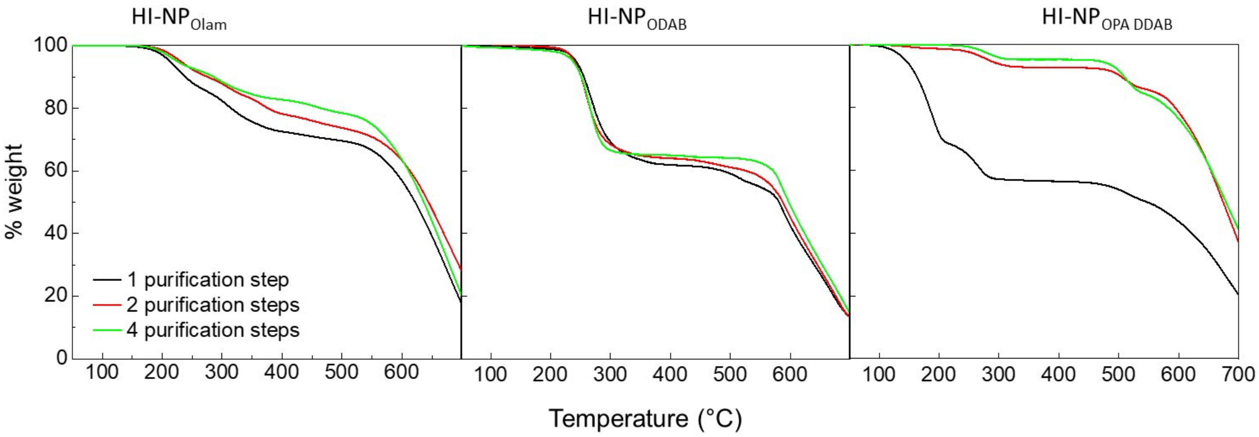

Figure S1. Thermogravimetric analysis of the three samples synthesized by hot-injection approaches after one (black like), two (red line) or four (green line) steps of purification by the addition of ethyl acetate to the reaction mixture or to the recovered pellet redispersed in toluene.

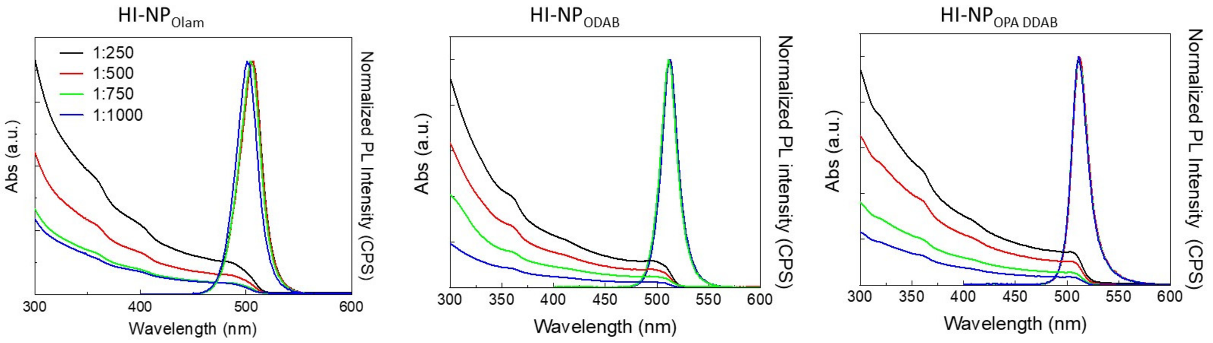

Figure S2. UV-Vis absorption and emission spectra (λ_{ex}=375 nm) of HI-NP$_{Olam}$, HI-NP$_{DDAB}$ and HI-NP$_{OPA DDAB}$ upon dilution.

1. Brown, A.A.M.; Vashishtha, P.; Hooper, T.J.N.; Ng, Y.F.; Nutan, G.V.; Fang, Y.; Giovanni, D.; Tey, J.N.; Jiang, L.; Damodaran, B., et al. Precise Control of $CsPbBr_3$ Perovskite Nanocrystal Growth at Room Temperature: Size Tunability and Synthetic Insights. *Chemistry of Materials* **2021**, *33*, 2387-2397, doi:10.1021/acs.chemmater.0c04569.
